# Male Moth Songs Tempt Females to Accept Mating: The Role of Acoustic and Pheromonal Communication in the Reproductive Behaviour of *Aphomia sociella*

**DOI:** 10.1371/journal.pone.0026476

**Published:** 2011-10-31

**Authors:** Jiří Kindl, Blanka Kalinová, Milan Červenka, Milan Jílek, Irena Valterová

**Affiliations:** 1 Institute of Organic Chemistry and Biochemistry, Academy of Sciences of the Czech Republic, Prague, Czech Republic; 2 Faculty of Electrical Engineering, Czech Technical University in Prague, Prague, Czech Republic; 3 Institute of Experimental Medicine, Academy of Sciences of the Czech Republic, Prague, Czech Republic; AgroParisTech, France

## Abstract

**Background:**

Members of the subfamily Galleriinae have adapted to different selective environmental pressures by devising a unique mating process. Galleriinae males initiate mating by attracting females with either chemical or acoustic signals (or a combination of both modalities). Six compounds considered candidates for the sex pheromone have recently been identified in the wing gland extracts of *Aphomia sociella* males. Prior to the present study, acoustic communication had not been investigated. Signals mediating female attraction were likewise unknown.

**Methodology/Principal Findings:**

Observations of *A. sociella* mating behaviour and recordings of male acoustic signals confirmed that males initiate the mating process. During calling behaviour (stationary wing fanning and pheromone release), males disperse pheromone from their wing glands. When a female approaches, males cease calling and begin to produce ultrasonic songs as part of the courtship behaviour. Replaying of recorded courting songs to virgin females and a comparison of the mating efficiency of intact males with males lacking tegullae proved that male ultrasonic signals stimulate females to accept mating. Greenhouse experiments with isolated pheromone glands confirmed that the male sex pheromone mediates long-range female attraction.

**Conclusion/Significance:**

Female attraction in *A. sociella* is chemically mediated, but ultrasonic communication is also employed during courtship. Male ultrasonic songs stimulate female sexual display and significantly affect mating efficiency. Considerable inter-individual differences in song structure exist. These could play a role in female mate selection provided that the female's ear is able to discern them. The *A. sociella* mating strategy described above is unique within the subfamily Galleriinae.

## Introduction

Moths of three main superfamilies (Pyralidoidea, Geometroidea, and Noctuidea) have tympanal ears sensitive to ultrasound [1], [2]. The ears probably developed in response to pressure from predators (bats) [3], [4]. Following ear development, many moth species developed sound producing organs and ultrasound emission became a part of sexual communication in some species [1], [5]–[7]. Members of the subfamily Galleriinae have adapted to different selective environmental pressures by devising an unusual mating system, where males initiate mating and attract females using either chemical or acoustic signals (Table 1). Three types of ultrasonic communication signals have been reported in Galleriinae to date: i) songs to attract mates (the lesser wax moth, *Achroia grisella*) [8]–[14], ii) courtship songs for mate acceptance/recognition (the greater wax moth, *Galleria mellonella* and the African sugarcane borer *Eldana saccharina* [15]–[20]), and iii) rival songs employed during male signalling (*A. grisella, G. mellonella, E. saccharina*) [9], [18], [21]–[23]. Ultrasonic emission in Galleriinae is associated with wing movements that induce high-frequency oscillations of tegular tymbals [1], [23]. The composition of male sex pheromones, tymbal morphology, and the sonic patterns, frequencies, and intensities of ultrasonic communication are all species-specific (Table 1).

**Table 1:** Literature data on premating communication in Galleriinae.

			Ultrasound				Male sex pheromone		
	Host (role)	Tegula	Frequency [kHz]	Intensity [dB]	Range [m]	Premating phase	Composition	Bioassay	References
**Galleriini**									
*Galleria mellonella*	*Apis mellifera* (parasite)	corrugated	72	81	unknown	courtship	9:Ald, 11:Ald, 9:OH, 11:OH, phytone	flight	[17], [18], [23], [46]
*Achroia grisella*	*Apis mellifera* (parasite)	corrugated	70–130	92	1–2	calling	11:Ald, Z11-18:Ald	walking	[8]–[12], [14], [25]
*Achroia innotata*	*Apis cerana*, *A. mellifera* (parasite)	not studied					9:Ald, 9:OH	contact	[47]
**Tirathabini**									
*Corcyra cephalonica*	rice (store pest)	striated	125	100	1–2	calling	farnesal (2 isomers)	walking	[23], [27], [29]
*Eldana saccharina*	sugar cane (pest)	striated	20–120 (peak 30–80)	unknown	unknown	unclear	eldanolide, 6,10,14-triMe-15:OH, 4-OH-benzaldehyde, vanilin	walking, flight	[20], [28], [48]
*Aphomia sociella* *	bumble bee (parasite)	striated	60–120 (peak 80–90)	102	0.02–0.4	courtship	2-phenylethanol, mellein, phytone, 2,6-nonadien-4-olide, 6-nonen-4-olide, 6:OH	flight	[24], [49], this paper
*Tirathaba mundella*	oil palm	not studied					2,2,6-triMe-6-vinyl-tetrahydropyran-3-ol, 6,10,14-triMe-15:OH, phytone, vanilin	walking, flight	[50]
*Paralipsa gullaris*	unknown (store pest)	not studied					2-phenylethanol, 2,6-nonadien-4-olide	contact	[51]
*Aphomia zelleri*	*Brachythecium albicans*, moss	not studied					odour of unknown composition	none	[52]

*Data for *Aphomia sociella* added from Results of this paper.

*Aphomia sociella L.* (Pyralidae, Galleriinae) is becoming an economically important pest in bumblebee mass-production facilities. Recently, six candidate compounds for the sex pheromone have been identified in the wing gland extracts and emanations of *A. sociella* males [24]. However, nothing is yet known about the signals that mediate female attraction in this species. The present study addresses the following questions: i) How do *A. sociella* males attract females? Do they use sex pheromones, acoustic signals, or a combination of the two? ii) What is the morphology of the sound-producing tymbals? iii) What are the physical parameters of acoustic signals (sonic patterns, frequencies, and intensities)? iv) How sensitive is the female ear? And, consequently, what is the communication range of ultrasonic signalling in *A. sociella*? v) Does ultrasonic communication affect mating efficiency? vi) How do our data fit into the evolution pattern of chemical and acoustic communication in Galleriinae?

## Materials and Methods

### 1. Insects

*Bombus terrestris* nests were colonised with *A. sociella* and left to overwinter. In the spring, larvae were allowed to pupate. After eclosion, the moths were segregated in individual plastic containers stoppered by moistened cotton plugs. Pilot experiments designed to determine the peak of mating activity were performed during the entire circadian period. Semi-field experiments were conducted after dusk. In the laboratory, the moths were kept in continuous light and used in experiments after 1 hour of acclimation in the dark. Virgin moths (2–4 days old) were utilised.

### 2. SEM of Tegular Morphology

Male and female tegulae were dissected and sonicated for 3 minutes to remove scales. The tegulae were then dehydrated in a series of 30–100% aqueous alcohol and 100% acetone. Dehydrated tegulae were mounted on aluminium stubs, coated with 300 nm of gold∶palladium (60∶40) alloy, and visualised using a JEOL 6380 LV scanning electron microscope.

### 3. *A. sociella* Mating Behaviour

Experiments designed to understand individual phases of the *A. sociella* mating behaviour were performed under red light and at ambient temperature/humidity. During photophase, pairs of insects were placed in individual round dishes (25 cm in diameter) made of wire mesh (one pair per dish). The same insects were then observed during scotophase. Ultrasonic signalling was detected using a bat detector (heterodyne Mini 3 Bat Detector, Ultra Sound Advice, UK) set at 80 kHz (the dominant frequency of *Aphomia sociella* ultrasonic songs – see bellow).

The role of ultrasonic signals in the mating process was studied in a subset of the above experiments by comparing the mating efficiency of intact males and males with removed tegulae (muted males). The tegulae were removed under CO_2_ narcosis with the aid of a stereomicroscope one day prior to the experiments. Control males were subjected to CO_2_ anaesthesia only. 70 pairs of moths were observed for each treatment and successful matings were counted. Collected data were statistically evaluated using a t-test.

### 4. Attractiveness of Intact Males and Dissected Pheromone Glands

The following set of experiments was designed to assess whether the male sex pheromone alone is sufficient to attract females. To that end, wing glands were dissected from males observed to wing fan for at least ten minutes. The males were chilled for 5 minutes after which their wing sex pheromone glands were dissected. Glands from two males (2 Male Gland Equivalents, 2MGEs) were placed on a filter-paper disc (Whatman No. 1, 1 cm in diameter) and gently crushed. The discs were mounted inside a cage, and the cage was suspended in a greenhouse (see bellow).

Greenhouse experiments were performed between 10 and 12 pm. Greenhouse dimensions were 3,2×1,2×2,0 m (LxWxH) with the tapered roof reaching 2,5 m. In these experiments, females were allowed to respond either to an intact male or to 2MGEs (referred to as “bait” from this point forward). The bait was placed inside a cage (round tea strainers made of wire mesh, 10 cm in diameter), and the cage was suspended at a height of 190 cm above ground (central axis, 200 cm from the release point – see bellow). The cage was illuminated by red light enabling observation. During the experiment, 20 females were released at the same time into the greenhouse from a single release point: central axis, height of 120 cm. The number of successful landings on the bait, the time elapsed from female release until landings, the amount of time spent on the bait, and any female sexual display (wing fanning) were recorded. Ultrasound generation was monitored by means of a bat detector. The females that have landed were removed 15 minutes after landing on the bait. In total, 100 females were subjected to each treatment (intact males or dissected glands) in five independent sessions. Each female was allowed to respond to only one treatment. Empty cages were used as controls. After the experiment, used cages were washed with hot water and detergent, rinsed with ethanol, and dried at 100°C. Collected data were statistically evaluated using a t-test.

### 5. Ultrasound Recording

To investigate the sound physical parameters and the role of ultrasonic signals in mating behaviour, real time sound recordings were obtained in the dark at ambient temperature and humidity. These experiments were performed inside an anechoic chamber (9.5×8.3×7.7 m) that eliminates external noise and echoes. The moths (placed individually inside wire cages, sexes separated) were allowed to acclimate in the chamber for 1 hour. During the acclimation period, males began to call (i.e. to wing fan and release pheromone). Cages with calling males were placed one at a time on a table equipped with recording instrumentation (see bellow). The behaviour of the males was observed under red light. Ultrasonic signals were recorded either during male calling, male courting (in the proximity of a female), or male rival behaviour (proximity to another male). Courting behaviour was initiated by the presence of a female (a female kept inside another cage was brought close to a cage containing a calling male). Rival behaviour was initiated by introducing a second male into a cage with a calling male.

Signals emitted by males were recorded using a 1/8″ Pressure Microphone GRAS Type 40DP (frequency range: ±2.0 dB from 6.5 Hz to 140 kHz). The recording instrument was placed next to cages containing calling males. GRAS Type 26AC Preamplifier and GRAS Type 12AK Power Module were used to amplify signals, and the signals were digitised by means of a computer plug-in NI PCI-6251 (National Instruments) multifunction data acquisition (DAQ) card at a sampling rate of 500 kS/s with 16-bit resolution (the DAQ card allows sampling at a maximum rate of 1.25 MS/s). The DAQ card was controlled by specially designed MothMaster software written in LabView (National Instruments). MothMaster allows data acquisition, conditioning (band-pass filtering), microphone calibration (in conjunction with Sound Calibrator GRAS Type 42AB), and data analysis in the time and frequency domains. The DAQ card used for ultrasound recording was equipped with two analogue channels (16-bit, max. 2.86 MS/s) that allowed playback experiments (see bellow).

### 6. Playback Experiments

The aim of playback experiments was to understand how ultrasound affects female behaviour and whether females are able to differentiate between male rival and courting songs. All experiments were performed inside an anechoic chamber under red light. Females were encaged individually in glass tubes (3 cm in diameter and 5 cm in length). The tubes were stoppered by a net nylon mesh held in place by a rubber band. Females enclosed in tubes were acclimated in the anechoic chamber for 1 hour. The tubes were then placed one at a time on a table with their stoppered ends oriented towards an ultrasonic loudspeaker. Played-back sounds were generated by MothMaster software that enables replay of previously recorded sounds. The replayed sounds were amplified by an S55 Amplifier and transmitted by means of an Ultra Sound Advice S56 loudspeaker (both manufactured by Ultra Sound Advice, UK). Sound intensity of the broadcast signals was SPL>85 dB at 0.25 m.

Prior to sound stimulation, each female was observed for 1 minute. After that, one chirp of a previously recorded courting or rival song was played on infinite repeat with a period of 2 seconds. The intensity of the acoustic signals was set to reach at most ∼100 dB peSPL at the location of the female. If a female did not start to wing fan within two minutes of initiation of ultrasound stimulation, she was classified as not responding. In a subset of playback experiments, the intensity of the replayed signals was systematically reduced to determine the hearing threshold of the females.

## Results

### 1. Tegular Morphology

**Figure 1:**
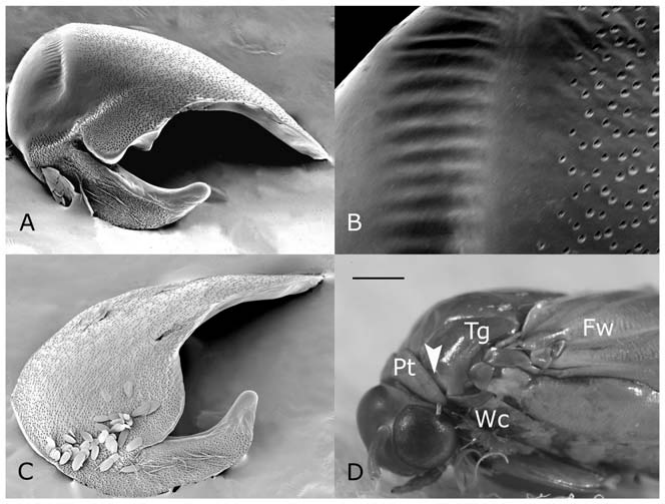
Scanning Electron Microscopy images of male and female tegulae (A,B,C) and stereomicroscopic photograph of male head and thorax (D). The figure shows that females lack tegular tymbals (C). Male tymbals (A) are located on the dorso-rostro-lateral tegular surfaces (D) and are transected by one narrow, regularly striated strip (A,B). Abbreviations: Pt – patagium, Wc – tegular wing coupler, Tg – tegula, Fw - forewing. The bar represents 100 µm in A, C, 200 µm in C, and 500 µm in D. Arrow depicts the striated strip.

*A. sociella* female tegulae lack tymbals (Fig. 1C), *i.e.* these females do not posses organs for ultrasound production. In males of the same species, the tymbals are situated dorso-laterally on the anterior part of the tegulae hidden under the patagium sclerite (Fig. 1A,D). The tymbal cuticle lacks scales and anteriorly forms a translucent-like area (arrow in Fig. 1D) with one transversally running strip of about 25 striae (Fig. 1A,B). Medially, the tegulae are associated with air chambers of the diaphragm (visible when the tegulae are removed, not shown).

### 2. *A. sociella* Mating Behaviour

Out of the 60 tested *A. sociella* pairs, 56 mated during the first half of scotophase. Males initiated mating by stationary wing fanning and pheromone dispersal (referred to as “calling” from now on). During calling, males did not wing fan continuously but in ∼1-second intervals interrupted by short rest periods (0.5 seconds on average). Consequently, the male pheromone plume has a pulse-like character. Wing fanning and pheromone dispersal are silent. The male pheromone has a pleasant floral odour and elicits positive chemo-tactic flying or walking responses in females. Approach by a female terminates male stationary wing fanning and triggers male courting behaviour characterized by walking, wing fanning, and especially ultrasonic signalling (courting songs). Females respond to male courting also by walking and wing fanning. Females don't produce ultrasound. In our experiment, receptive females were observed to adopt a special position that allowed males to attempt copulation. Mating took place shortly thereafter. Non-receptive females were abandoned after repeated unsuccessful courting; rejected males often began to call again.

Males also emitted ultrasound when another male was introduced into their cage. If the introduced male interfered with the calling of the original resident, the resident stopped calling, approached the intruder, produced ultrasonic songs, and physically assaulted the intruder. The intruder responded in a similar manner. After a while, both males took up new positions within the available territory and eventually started to call again. This antagonistic behaviour with ultrasonic signalling was observed in 15 of the 30 investigated male pairs. The antagonistic behaviour was more pronounced when a female (or her odour) was present (usually a cage containing a female and placed next to the observation point was sufficient to increase the intensity of the described antagonistic behaviour).

As part of both courting and competing behaviours, males produced ultrasound while simultaneously walking and wing fanning. No visible changes in wing position associated with ultrasonic signalling were observed (videotape analysis). Muted males were significantly less successful in mating than intact males (Table 2). In a no-choice test (experiments with pre-selected pairs), we recorded 9.43±0.79 copulations of intact males while only 3.9±0.69 copulations of muted males (n = 7, N per replicate = 10). However, females mated equally well with intact males as with males with one tegula removed (songs of semi-muted males could not be differentiated from songs of intact males; Table 2).

**Table 2:** Mating effectiveness of intact, muted, and semi-muted (one tegula removed) males.

	Number of copulations [X±SEM], choice test	
Intact/muted males	7.2±0.37/2.8±0.37	Significant
Intact/semi-muted males	5.2±0.2/4.8±0.2	Non-significant

Significance was evaluated by t-test, P 0.05. Values represent means per replicate (n = 7, N per replicate = 10) and SEM.

To summarise, the reproductive behaviour of *A. sociella* has 5 phases: calling (male wing fanning and pheromone dispersal), female approach, courting (male ultrasonic signalling), female sexual display (female wing fanning and pheromone dispersal), and mating.

### 3. The Attractiveness of Sex Pheromone

**Figure 2:**
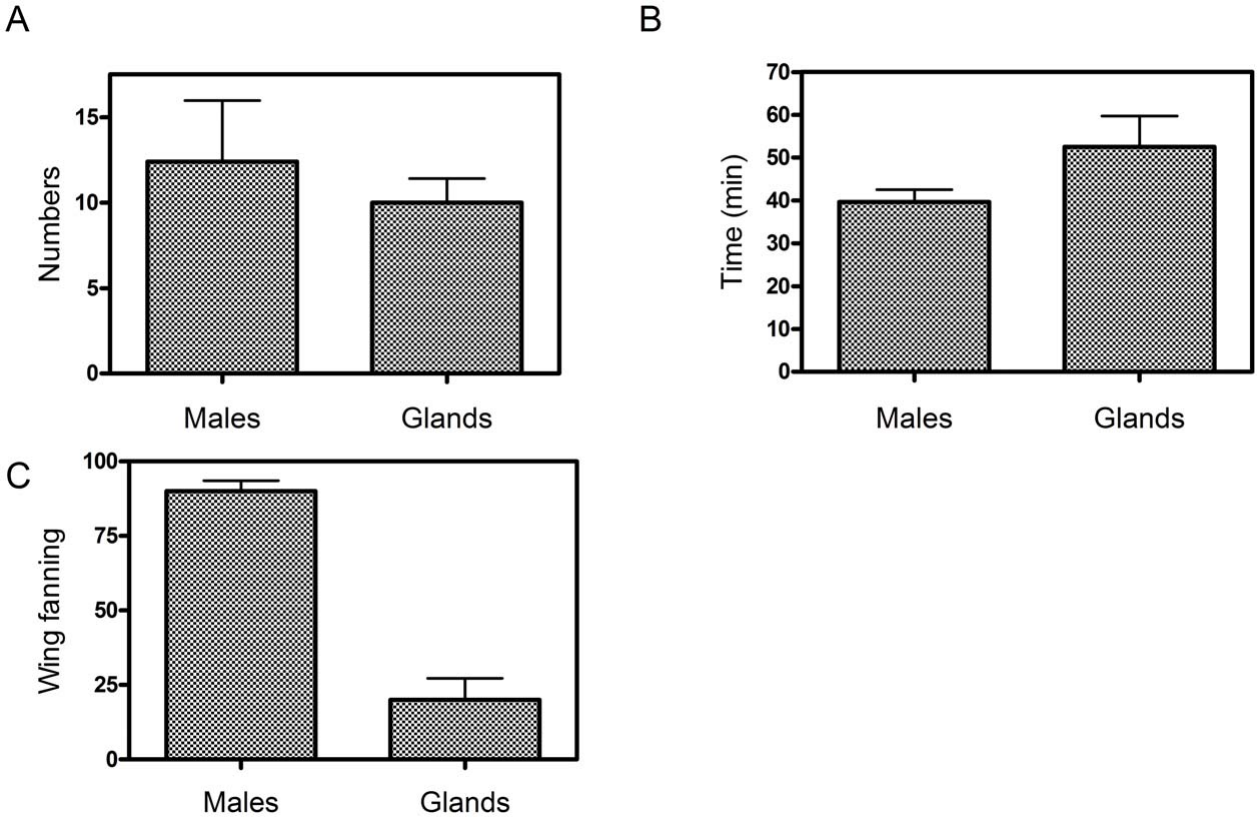
A comparison of the attractiveness of *A. sociella* calling males and the gland extracts (two male gland equivalents, 2 MGEs). Fig. **2A** shows the number of females attracted by one intact male or two pairs of excised male wing glands; Fig. **2B** shows the time required by searching females to find calling intact male and 2 MGEs; Fig. **2C** shows the percentage of females that wing fan on the baited cage in response to intact male or to 2MGEs. Only 20% of attracted females remain arrested on the cage and wing fan in response to male glands.

In greenhouse experiments, dissected male glands were nearly as attractive as intact calling males (Fig. 2A,B). Intact males and 2MGEs attracted 62 and 50 females, respectively, out of a total number of 100 females tested for each treatment (Fig. 2A). The observed difference was not statistically significant (t-test, p = 0.05, N = 100). Females responding to the 2MGEs required a slightly longer but statistically insignificant (at p = 0.05 level) time (52 min) to localize the calling source than did females responding to intact males (39 min) (Fig. 2B). Females responding to intact males remained considerably longer on the cage (5–10 min on average) than did females responding to dissected glands (less than 1 min) (Fig. 2C). Females responded to courting intact males by walking on the surface of the cage and wing fanning. Females responding to dissected glands remained on the cage for a shorter period of time, wing fanned less frequently (Fig. 2C, differences are statistically significant), and usually flew away shortly after landing. Landing of a female on the cage always terminated male calling and initiated male courting behaviour.

To summarize, our data show that male pheromone alone is sufficient to mediate female attraction.

### 4. Sound Analysis and Playback Experiments

**Figure 3:**
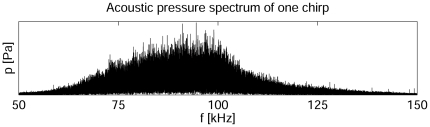
Acoustic power spectrum of one chirp.

*A. sociella* males emit relatively strong ultrasonic signals. The maximum peak sound intensity recorded in our experiments reached ∼102 dB peSPL (20 µPa) at a distance of 5 cm. The majority of the acoustic energy of male songs was transmitted within a frequency range of ∼60 kHz to ∼120 kHz with a maximum at ∼80–90 kHz (Fig. 3). A few studied specimens operated within a frequency range shifted slightly upward or downward ranging from ∼50 kHz to ∼140 kHz (not shown).

**Figure 4:**
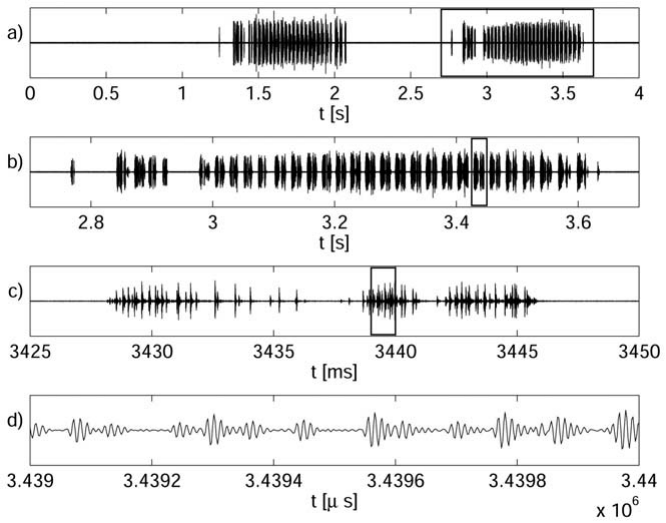
The time course of the male's ultrasonic courting song. a) two chirps separated by a silent period, b) a detail of one chirp, c) a detail of one modulation cycle, d) individual clicks.

**Figure 5:**
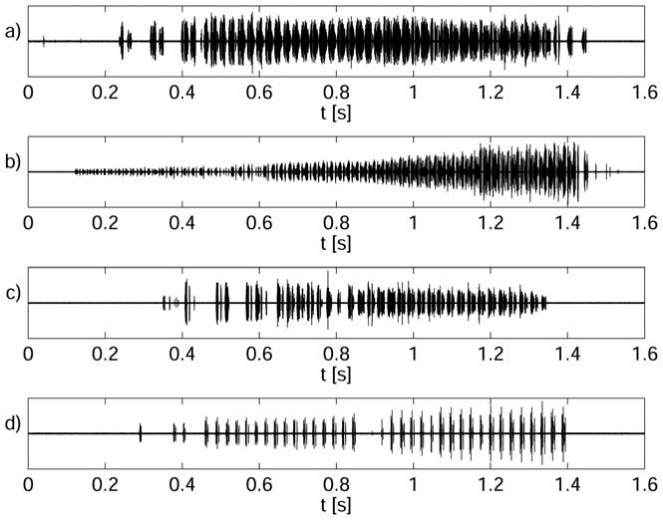
The individual variability (time courses) in male song chirps a,b) mating chirps; c,d) are rival chirps.

Male ultrasonic signals consisted of chirps (Figs. 4a) separated by highly irregular intervals of silence (mean ± standard deviation of the mean 1540±120 ms, N = 84 samples, 8 males). The duration of courting and rival song chirps was found to differ slightly. The average duration of courting and rival songs was 1122±17 ms (11 males, N = 110, Gaussian-like distribution) and 1050±20 ms (6 males, N = 66, Gaussian-like distribution), respectively (t-test, *p* = 0.004). Both courting and rival chirps consisted of individual modulation cycles (MCs) (Fig. 4b). The average period of a courting MC was 26.2±0.3 ms (10 males, N = 27, Gaussian-like distribution), whereas it was 26.7±0.4 ms for a rival MC (6 males, N = 18, Gaussian-like distribution). The difference between the duration of the modulation cycles of rival and courting songs was not statistically significant (t-test, *p* = 0.32). Both chirp types often began with a few MCs possessing a longer time separation interval (Fig. 4b). MCs were composed of trains of short ultrasonic pulses (clicks) (Fig. 3d), the duration of which varied within an interval from ∼50 µs to ∼100 µs and often overlapped. The number and distribution of individual clicks in an MC (its structure) varied substantially from one specimen to the next, but the chirps of individual males were very consistent (Fig. 5). Figure 5 shows examples of variability (time courses) in the structure of male mating chirps (Fig. 5a,b) and male rival chirps (Fig. 5d,e).

Playback experiments in an anechoic chamber revealed that females did not discriminate between courting and rival songs. Both ultrasonic signals elicited female wing fanning. Out of twenty females exposed to a courting song, ten immediately started wing fanning, four wing fanned after 5 seconds, and two wing fanned after 30 s of playback. Only four females did not respond at all to ultrasonic signals. Similarly, in the presence of a rival song, ten females immediately started to fan their wings, three started after 10 seconds, and three after 20 seconds of playback. Four females did not respond. These experiments show that acoustic signals alone are sufficient to elicit female wing fanning behaviour. The presence of other stimuli (chemical, optical, mechanical) is not required. Both courting and rival songs invoked female wing fanning behaviour equally well. The playback experiments also revealed that 13 out of 15 females responded by wing fanning to replayed male chirps with a peak sound intensity of 92 dB at the female's position, while only 4 out of 15 females responded to 82 dB. No females responded to sound with an intensity of 72 dB. We can thus conclude that the threshold level of an ultrasonic signal for triggering a female's response lies in the vicinity of 82 dB.

To summarise, females responded similarly (by wing fanning) to male courtship and rival songs.

## Discussion

Our data show that *A. sociella* males initiate mating by means of a sex pheromone released from their wing glands and dispersed by wing fanning. Acoustic signals mediate courtship interactions. Pair forming sequence follows these steps: Males attract females by a sex pheromone released during calling (advertisement signaling). Females fly towards the calling males. The males become aware of a female's presence prior to her wing fanning (sexual arousal). The presence of a female triggers male ultrasonic communication, which in turn “attracts the female's attention”, stimulates her sexual arousal, and facilitates her receptivity to courting. The mating system of *A. sociella* thus resembles those of *G. mellonella* [15]–[18] and *E. saccharina* [20] and differs from those of *A. grisella* [8]–[13], [21], [25] and *C. cephalonica* [26]–[30]. Contrary to other Galleriinae species, where female moths walk rather than fly (*C. cephalonica* [29], *E. saccharina* [20], [28], *A. grisella* [8]–[11], *G. mellonella* [31]), *A. sociella* female moths fly towards calling males.

Galleriinae males tend to aggregate in the vicinity of larval food sources, call jointly, and display rival behaviour in the presence of a female [15], [25], [32]. We do not yet know where *A. sociella* pairs mate, but our observations from the greenhouse suggest that *A. sociella* males may tend to aggregate and call jointly as well. Similarly to other Galleriinae species, *A. sociella* males display rival behaviour associated with ultrasonic signalling in the presence of a female. Observed courting and rival signals were similar in structure and triggered sexual display in females equally well. Ultrasonic chirp structures of courting and rival songs were the same for a given individual but differed among individual males. Such variability reflects unique differences that might represent the basis for female sexual selection [9], [13], [33]–[35] provided that the variability is functional, i.e. that *A. sociella* females can detect it.

Our study found that male tymbals in *A. sociella* are striated, resemble those found in *C. cephalonica* [27] and *E. sacharina* [20], [36], and differ from the finely corrugated tymbals of *G. mellonella* [15], [25] and *A. grisella* [15]. The mechanism of sound production in *A. sociella* is not known, but we hypothesize that it resembles that of other Galleriinae species [16], [25], [37]. However, the alternating ultrasound and silent periods seen during *A. sociella* male ultrasonic production was not associated with observable changes in wing position during fanning (to switch the ultrasonic production on and off) as described in *A. grisella* [25], for instance.

Both the intensity of ultrasonic signals of *A. sociella* males (Table 1) and the hearing threshold of *A. sociella* females (82 dB) were slightly higher than those of other Galleriinae species. The hearing thresholds of *A. grisella* and *G. mellonella* are estimated at 50–82 dB and 69 dB, respectively (review [38]). The intensity of courting signals for other Lepidoptera outside of the subfamily Galleriinae is also lower [6], [39]. The higher intensity of ultrasonic signals in *A. sociella* and the lower threshold of hearing in the females of this species may reflect species-specific ecological adaptations.

Considering the hearing threshold of *A. sociella* females and the drop-off in sound intensity due to atmospheric attenuation (2.7 dB/m at 90 kHz) [40], we expect the maximum range for ultrasonic communication in this species to be 0.45 m. This maximum theoretical value is consistent with our behavioural observations; all *A. sociella* females responded by wing fanning to courting males at a distance of 0.25 m (N = 10).

Our study provided evidence that ultrasound signalling in *A. sociella* is triggered only when other conspecifics are nearby. The triggering signals operate in the dark and at close range (cm). Direct contact between the moths is not necessary [41]. Both chemical and mechanical signals may be involved. Our experiments with freshly frozen moths [41] revealed that motionless males and females both triggered ultrasonic signalling in *A. sociella* males. However, frozen females were more efficient in triggering male ultrasonic signalling than frozen males: while only 4 out of 20 males responded to another dead male, all 20 males responded to dead females [41]. These experiments suggest that chemical signals play a role in both intra-sexual and inter-sexual communication. Freshly frozen males may emanate sex pheromones that can be smelled by another male and registered by male antennae bearing olfactory receptors sensitive to pheromone gland emanations [24]. The fact that males were much more sensitive to dead females than to dead males led to the discovery of female-specific sex pheromone in *A. sociella* [41]. Female-specific sex pheromone was also reported in *C. cephalonica* [29], [30]. Apart from pheromones, mechanical signals may contribute to the ability of *A. sociella* males to detect females from a distance. Unsteady flow of air associated with wing fluttering [42] or mechanical vibrations of a substrate after female landing [23] may also play a role.

Ultrasonic signals produced by *A. sociella* males fall within the range of bat echolocation. It would be interesting to study whether *A. sociella* females are able to discriminate between bat and intraspecific ultrasonic songs (as is the case in *G. mellonella* [43]).

Our experiments show that ultrasonic signalling significantly affects *A. sociella* mating efficiency. A similar phenomenon was recently described in *O. furnacalis* [44] and several other moth species ([6], [7] and citations therein). A comparison of the premating behaviour of *A. sociella* with that of other Galleriinae species (Table 1) reveals that both courtship characteristics and pheromone chemistry correlate poorly with taxonomic relationships. This suggests that the selective pressures governing the evolution and maintenance of courtship were distinct from those involved in the evolution of other morphological characteristics. A strong relationship between mating strategies and resource structures (spatial distribution of larval food) has been suggested for Lepidoptera [38], [45]. In concert with this theory, the non-conform mating strategy in Galleriinae would have been expected to evolve in response to patchy and temporary larval habitats. The pheromonal and sonic signals of males calling from locations rich in resources may enhance the habitat's attractiveness to females and thus increase their chances of finding larval food [45]. Further experiments are needed, however, to determine whether the *A. sociella* mating system is also resource-based.

In conclusion, we found that *A. sociella* males attract females by means of a sex pheromone. Male ultrasonic signalling is triggered by the presence of a female. Ultrasonic signals initiate courtship, stimulate female sexual display, and significantly affect mating efficiency. This mating strategy is unique within the subfamily Galleriinae as compared to known strategies of other related moths. However, courtship songs that prompt females to accept mating are known in some moth species with “normal” (female-initiated) mating systems.
